# Small Intramucosal Sequestra in Medication-Related Osteonecrosis of the Jaw: A Case Series

**DOI:** 10.3390/diagnostics16172731

**Published:** 2026-08-26

**Authors:** Usagi Hayashi, Gaku Koizumi, Kenya Okumura, Rina Yamada, Kokoro Nagata, Kazuto Kurohara, Taku Murata, Kasumi Shimizu, Akinobu Hayashi, Keiichi Yamanaka, Naoya Arai

**Affiliations:** 1 Department of Oral and Maxillofacial Surgery, Mie University Graduate School of Medicine, 2-174 Edobashi, Tsu City 514-8507, Mie, Japan; hayashiu8843@med.mie-u.ac.jp (U.H.); k-gaku@med.mie-u.ac.jp (G.K.); yamada-r@med.mie-u.ac.jp (R.Y.); muratat@med.mie-u.ac.jp (T.M.);; 2 Pathology Division, Mie University Hospital, 2-174 Edobashi, Tsu City 514-8507, Mie, Japan; 3 Department of Dermatology, Mie University Graduate School of Medicine, 2-174 Edobashi, Tsu City 514-8507, Mie, Japan

**Keywords:** medication-related osteonecrosis of the jaw, antiresorptive agents, oral mucosa, intramucosal sequestra, pseudoepitheliomatous hyperplasia

## Abstract

**Background/Objectives:** Medication-related osteonecrosis of the jaw (MRONJ) is a known side effect of antiresorptive agents. Although the jawbone has been extensively investigated, histopathological changes in the overlying oral mucosa remain poorly characterized. We previously reported small bone fragments located within the oral mucosa of a patient with MRONJ. This study aimed to investigate the presence and histopathological characteristics of those fragments in other patients with surgically treated MRONJ. **Case Presentation:** This retrospective case series included six patients with stage 3 mandibular MRONJ who underwent marginal or segmental resection. Archived histological sections from these patients were examined. Oral mucosal specimens had been separated from the underlying osseous lesions for independent histological evaluation. In this study, small intramucosal sequestra were defined as small bone fragments that appeared to be completely surrounded by mucosal connective tissue in individual two-dimensional sections. All sections had been stained with hematoxylin and eosin, and additional von Kossa staining had been performed on oral mucosal sections from five of the six cases. Histopathological evaluation of small intramucosal sequestra included the presence, size range, and association with pseudoepitheliomatous hyperplasia (PEH). **Results:** Small intramucosal sequestra were identified in all six patients. They were present either singly or in clusters within the connective tissue beneath the oral epithelium. The intramucosal sequestra exhibited empty osteocytic lacunae, indicating devitalized bone, and were positive for von Kossa staining in all five examined cases, confirming their mineralized nature. In two patients, PEH was observed in direct contact with the small sequestra, some of which appeared to be embedded within the advancing epithelial fronts. **Conclusions:** Small intramucosal sequestra were a recurrent histopathological finding in this selected series of surgically resected stage 3 mandibular MRONJ. Their presence highlights histopathological changes in the oral mucosa overlying necrotic jawbone that have received limited attention. Further studies including broader patient populations and appropriate comparison groups are needed to determine the generalizability and disease specificity of this finding and its potential relevance to MRONJ pathogenesis.

## 1. Introduction

Medication-related osteonecrosis of the jaw (MRONJ) is a serious adverse event associated with antiresorptive agents, including bisphosphonates and denosumab. The precise mechanisms underlying its development remain incompletely understood. Because antiresorptive agents profoundly suppress bone remodeling, the jawbone has long been regarded as the primary site of pathological change in MRONJ [1,2,3]. Histopathological studies of osseous lesions have demonstrated necrotic bone characterized by empty osteocytic lacunae, inflammatory changes including acute or chronic osteomyelitis, and frequent colonization by Actinomyces species [4,5,6,7].

In contrast, histopathological changes in the oral mucosa overlying MRONJ lesions have received much less attention. This is partly because surgical specimens from patients with MRONJ usually consist predominantly of bone tissue and often contain only limited amounts of well-preserved soft tissue suitable for detailed histological evaluation. Consequently, reported mucosal findings have been largely limited to nonspecific inflammatory changes, including inflammatory cell infiltration, granulation tissue formation, fibrosis, and reactive epithelial proliferation in the form of pseudoepitheliomatous hyperplasia (PEH) [6,7,8,9,10].

During extensive surgical treatment of MRONJ, oral mucosa adjacent to the necrotic bone can be excised together with the underlying lesion, providing well-preserved soft tissue for histopathological examination. Using oral mucosal tissue obtained during such surgery, we previously identified multiple von Kossa-positive devitalized bone fragments within the subepithelial connective tissue of the oral mucosa. These small intramucosal sequestra exhibited empty osteocytic lacunae, and some were closely associated with the advancing epithelial fronts of PEH, suggesting a possible interaction between devitalized bone fragments and the overlying oral epithelium [11].

Therefore, the present retrospective case series investigated the presence and histopathological characteristics of small intramucosal sequestra in oral mucosal specimens obtained from six patients with surgically treated stage 3 mandibular MRONJ.

## 2. Materials and Methods

### 2.1. Ethics Statement

This retrospective case series was conducted in accordance with the Declaration of Helsinki and approved by the Ethics Committee of Mie University Hospital (Approval No. H2024-061).

### 2.2. Patients

Six patients who underwent surgical treatment for medication-related osteonecrosis of the jaw (MRONJ) at Mie University Hospital between January 2022 and December 2024 were included in this study. These were the only six patients with MRONJ who underwent surgical treatment during this period at our hospital. All six were primary surgical cases and had no history of any previous surgical intervention involving the jawbone whether related to MRONJ or other conditions. The diagnosis of MRONJ was established according to the diagnostic criteria provided in the 2022 update of the American Association of Oral and Maxillofacial Surgeons (AAOMS) position paper.

Clinical information, including age, sex, underlying disease, antiresorptive medication, duration of therapy, lesion site, stage, and surgical procedure (marginal or segmental resection), was obtained from the medical records.

### 2.3. Tissue Preparation

Immediately after surgical resection, the oral mucosal tissue and jawbone were carefully separated using a mucoperiosteal elevator to enable independent histopathological evaluation. Oral mucosal specimens were processed without decalcification, whereas bone specimens were decalcified with ethylenediaminetetraacetic acid (EDTA). All specimens were fixed in 4% phosphate-buffered formalin, embedded in paraffin according to the standard protocols of the Department of Pathology, Mie University Hospital, and 5-μm sections were prepared.

For this retrospective study, archived histological sections were retrieved and reviewed. The number of paraffin blocks prepared for each patient, the intervals between sections, and further details regarding tissue sampling could not be determined from the available records. All sections had been stained with hematoxylin and eosin (H&E), and von Kossa staining had also been performed on oral mucosal sections from five of the six cases.

Only sections in which both the oral epithelium and the underlying connective tissue were sufficiently well preserved for histopathological evaluation were included in the analysis.

### 2.4. Histopathological Evaluation

Histopathological evaluation focused primarily on the oral mucosa overlying the osseous lesion. The presence of inflammatory cell infiltration, PEH, and small intramucosal sequestra was assessed in each section. Bone specimens were examined for osteomyelitis, necrotic bone characterized by empty osteocytic lacunae, and Actinomyces colonies.

Small intramucosal sequestra were defined as devitalized bone fragments exhibiting empty osteocytic lacunae that appeared to be completely surrounded by the connective tissue of the oral mucosa in individual two-dimensional histological sections. Bone fragments exposed on the mucosal separation surface were excluded because they could have been mechanically introduced during separation of the oral mucosa from the underlying jawbone. Accordingly, only bone fragments that appeared to be completely embedded within and surrounded by the connective tissue in histological sections were included as small intramucosal sequestra for analysis.

For each case, the presence or absence of small intramucosal sequestra and the size range of the identified sequestra were recorded. In addition, the relationship between small intramucosal sequestra and PEH was evaluated by assessing whether direct contact was present between the advancing epithelial fronts of PEH and the sequestra. The presence of small intramucosal sequestra was confirmed by one pathologist and two experienced oral and maxillofacial surgeons at Mie University Hospital. Microphotographs were captured using a light microscope (ECLIPSE Si; Nikon Solutions Co., Ltd., Tokyo, Japan) equipped with a digital camera (Digital Sight 1000; Nikon Solutions Co., Ltd.). The longest diameters of the sequestra were measured using the built-in measurement software of the camera.

Because this study was designed as a descriptive case series, no statistical analyses were performed.

## 3. Results

The clinical characteristics of the six patients are summarized in Table 1. The patients comprised two men and four women, ranging in age from 61 to 87 years. Antiresorptive therapy consisted of bisphosphonates in four patients and denosumab in two patients, with treatment durations ranging from 1 to 6 years. All patients had stage 3 MRONJ involving the mandible and underwent either marginal or segmental resection. Patient 1 in Table 1 was a previously reported patient in our earlier single-case study [11].

**Figure 1:**
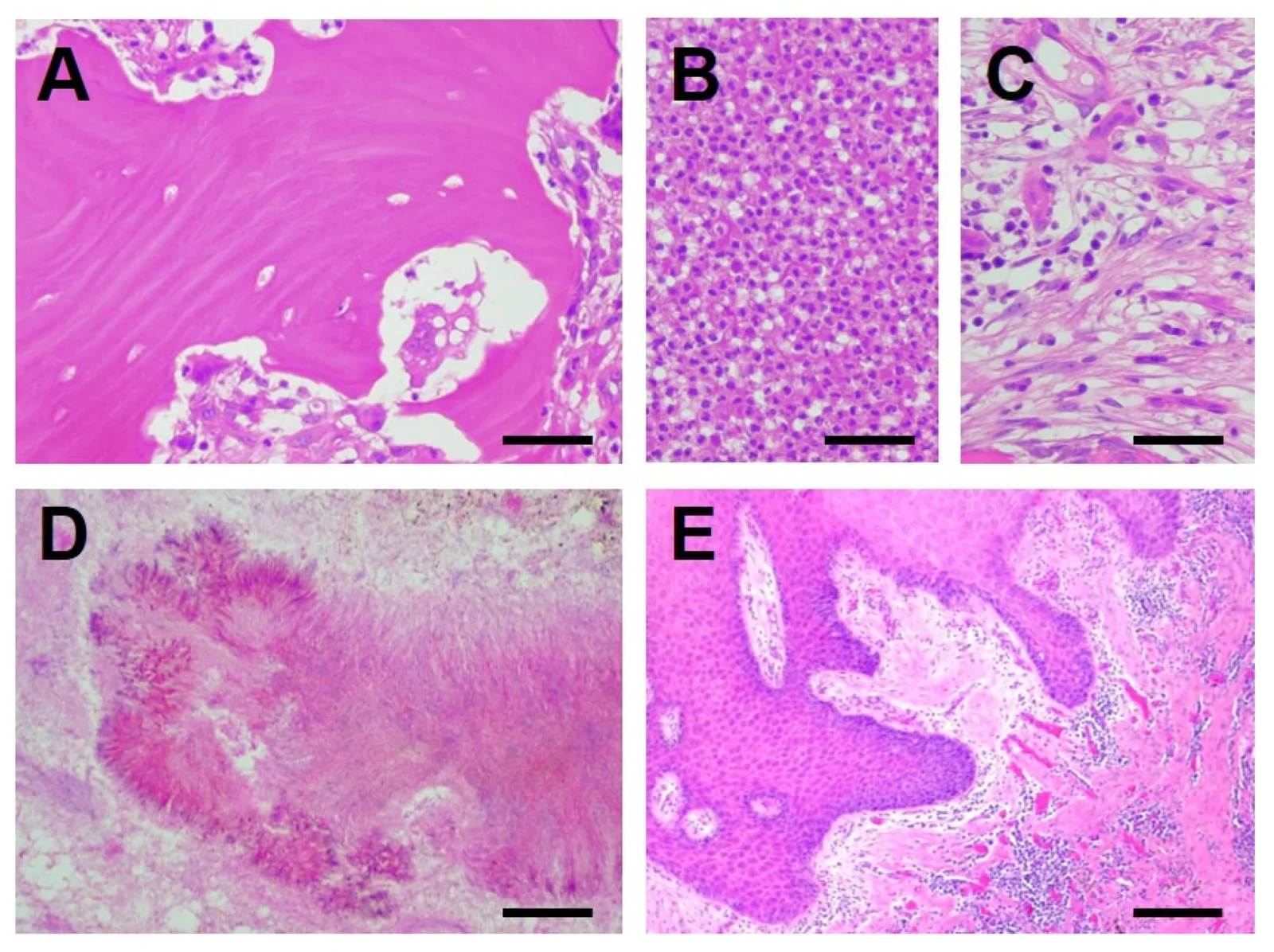
Representative histopathological findings in bone specimens (**A**–**D**) and oral mucosal specimens (**E**). Panels (**A**–**E**) are from patient 3 in Table 1. Panel (**A**) shows necrotic trabecular bone with empty osteocytic lacunae. Panels (**B**,**C**) show bone marrow spaces occupied by inflammatory cells (**B**) and fibrous tissue (**C**), respectively. Panel (**D**) shows colonies of Actinomyces. Panel (**E**) shows pseudoepitheliomatous hyperplasia (PEH) associated with inflammatory cell infiltration. Hematoxylin and eosin staining. Original magnification: ×200 (**A**–**D**) and ×40 (**E**). Scale bars: 50 μm (**A**–**D**) and 200 μm (**E**).

Histopathological examination of the bone specimens demonstrated necrotic bone with empty osteocytic lacunae (Figure 1A) and findings consistent with osteomyelitis in all patients. Bone marrow spaces were occupied by inflammatory cells (Figure 1B) in some areas and by fibrous connective tissue (Figure 1C) in others, corresponding to acute and chronic osteomyelitis, respectively. Colonies of Actinomyces were identified in three of the six patients (Figure 1D). Histopathological examination of the oral mucosal specimens demonstrated inflammatory cell infiltration in all patients together with PEH (Figure 1E).

**Figure 2:**
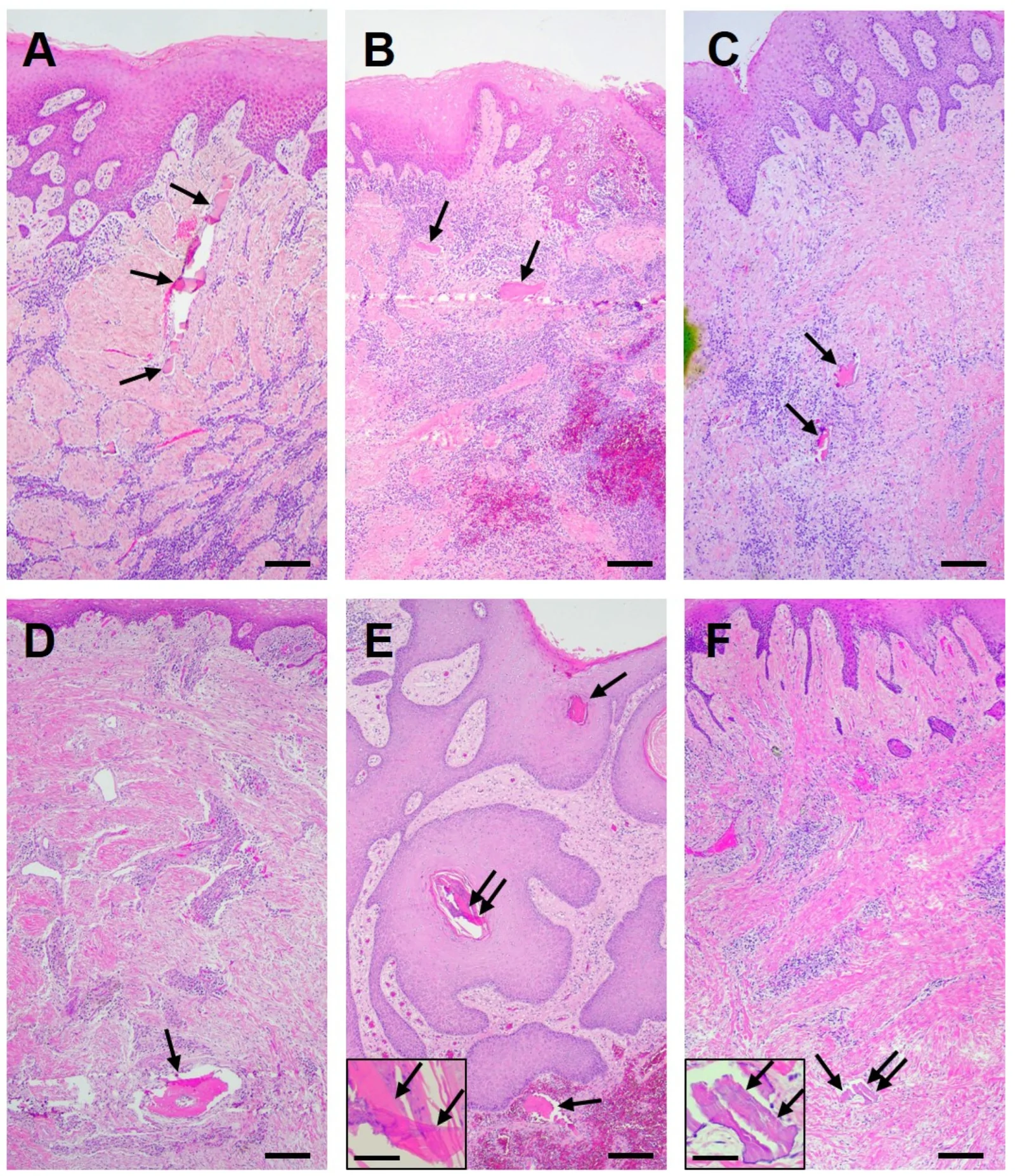
Small intramucosal sequestra identified in all six patients with medication-related osteonecrosis of the jaw (MRONJ). Representative photomicrographs from each of the six patients are shown (**A**–**F**). Panels (**A**–**F**) correspond to patients 1–6 in Table 1, respectively. Arrows indicate small intramucosal sequestra. Insets in panels (**E**,**F**) show high-magnification views of the small sequestra indicated by double arrows. The spaces around the sequestra are processing artifacts resulting from sectioning without decalcification. Hematoxylin and eosin staining. Original magnification: ×40 (**A**–**F**) and ×200 (insets). Scale bars: 200 μm (**A**–**F**) and 50 μm (insets).

Small intramucosal sequestra were identified in all six patients. The numbers of evaluable oral mucosal sections were 15, 10, 10, 8, 8, and 4 for patients 1–6, respectively. Small intramucosal sequestra were not detected in every evaluable section but were identified in 9/15, 3/10, 2/10, 3/8, 4/8, and 2/4 sections, respectively. They were present either singly or in clusters within the subepithelial connective tissue and ranged in size from 45 to 840 μm (Figure 2).

**Figure 3:**
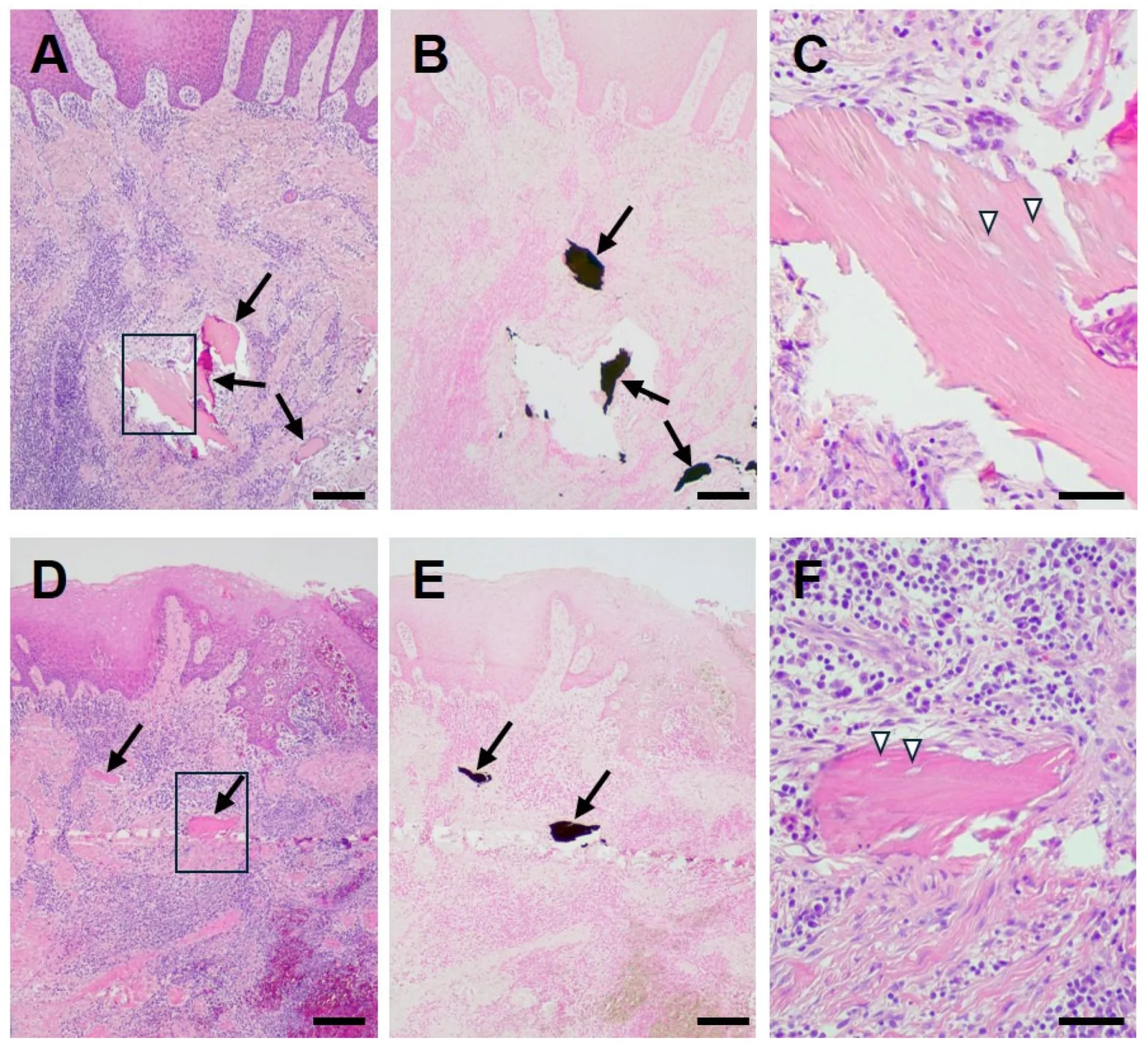
Histopathological characteristics of small intramucosal sequestra in patients with medication-related osteonecrosis of the jaw (MRONJ). Panels (**A**–**C**) are from patient 1 in Table 1, and panels (**D**–**F**) are from patient 2 in Table 1. Panels (**B**,**E**) are serial sections corresponding to panels (**A**,**D**), respectively. Panels (**C**,**F**) show high-magnification views of the boxed areas in panels (**A**,**D**), respectively. Arrows indicate small intramucosal sequestra, and arrowheads indicate empty osteocytic lacunae. The spaces around the sequestra are processing artifacts resulting from sectioning without decalcification. Hematoxylin and eosin staining (**A**,**C**,**D**,**F**) and von Kossa staining (**B**,**E**). Original magnification: ×40 (**A**,**B**,**D**,**E**) and ×200 (**C**,**F**). Scale bars: 200 μm (**A**,**B**,**D**,**E**) and 50 μm (**C**,**F**).

The small intramucosal sequestra exhibited empty osteocytic lacunae and showed positive von Kossa staining in all examined cases, confirming their mineralized nature (Figure 3). In most sections, the small sequestra appeared completely or partially detached from the surrounding connective tissue. The spaces around the sequestra were interpreted as processing artifacts resulting from sectioning without decalcification.

**Figure 4:**
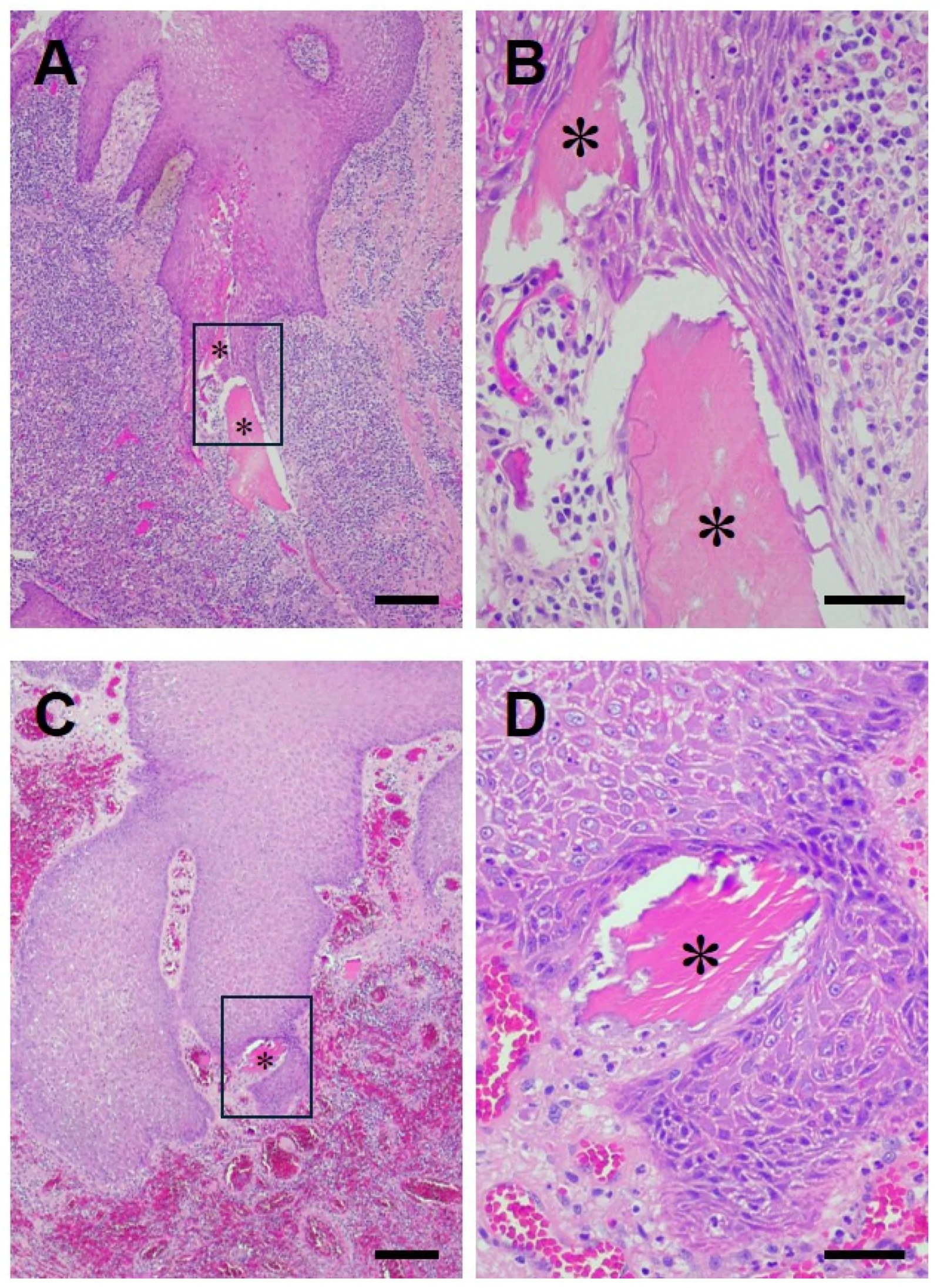
Photomicrographs showing small intramucosal sequestra adjacent to the advancing epithelial fronts of pseudoepitheliomatous hyperplasia (PEH). Panels (**A**,**B**) are from patient 1 in Table 1, and panels (**C**,**D**) are from patient 5 in Table 1. Panels (**B**,**D**) show high-magnification views of the boxed areas in panels (**A**,**C**), respectively. Asterisks indicate small intramucosal sequestra. The spaces around the sequestra are processing artifacts resulting from sectioning without decalcification. Hematoxylin and eosin staining. Original magnification: ×40 (**A**,**C**) and ×200 (**B**,**D**). Scale bars: 200 μm (**A**,**C**) and 50 μm (**B**,**D**). The upper sequestrum in panel (**B**) and the sequestrum in panel (**D**) are located within the hyperplastic epithelium.

Direct contact between small intramucosal sequestra and the advancing epithelial fronts of PEH was observed in two patients (Figure 4). In these cases, some sequestra were located adjacent to the advancing epithelial fronts, whereas others appeared to be located within the hyperplastic epithelium.

The histopathological findings in the oral mucosal specimens are summarized in Table 2.

## 4. Discussion

The present case series extends our previous single-case report by demonstrating that small intramucosal sequestra were identified in all six patients with Stage 3 MRONJ. Previous histopathological studies of MRONJ have focused primarily on osseous lesions, whereas the oral mucosa has generally been considered to show only nonspecific inflammatory changes. In contrast, the present study highlights the oral mucosa as an important site of pathological changes associated with MRONJ. Although the size and distribution of small intramucosal sequestra varied among patients, this study suggests that they represent a recurrent histopathological finding in this selected series of surgically treated Stage 3 mandibular MRONJ.

**Figure 5:**
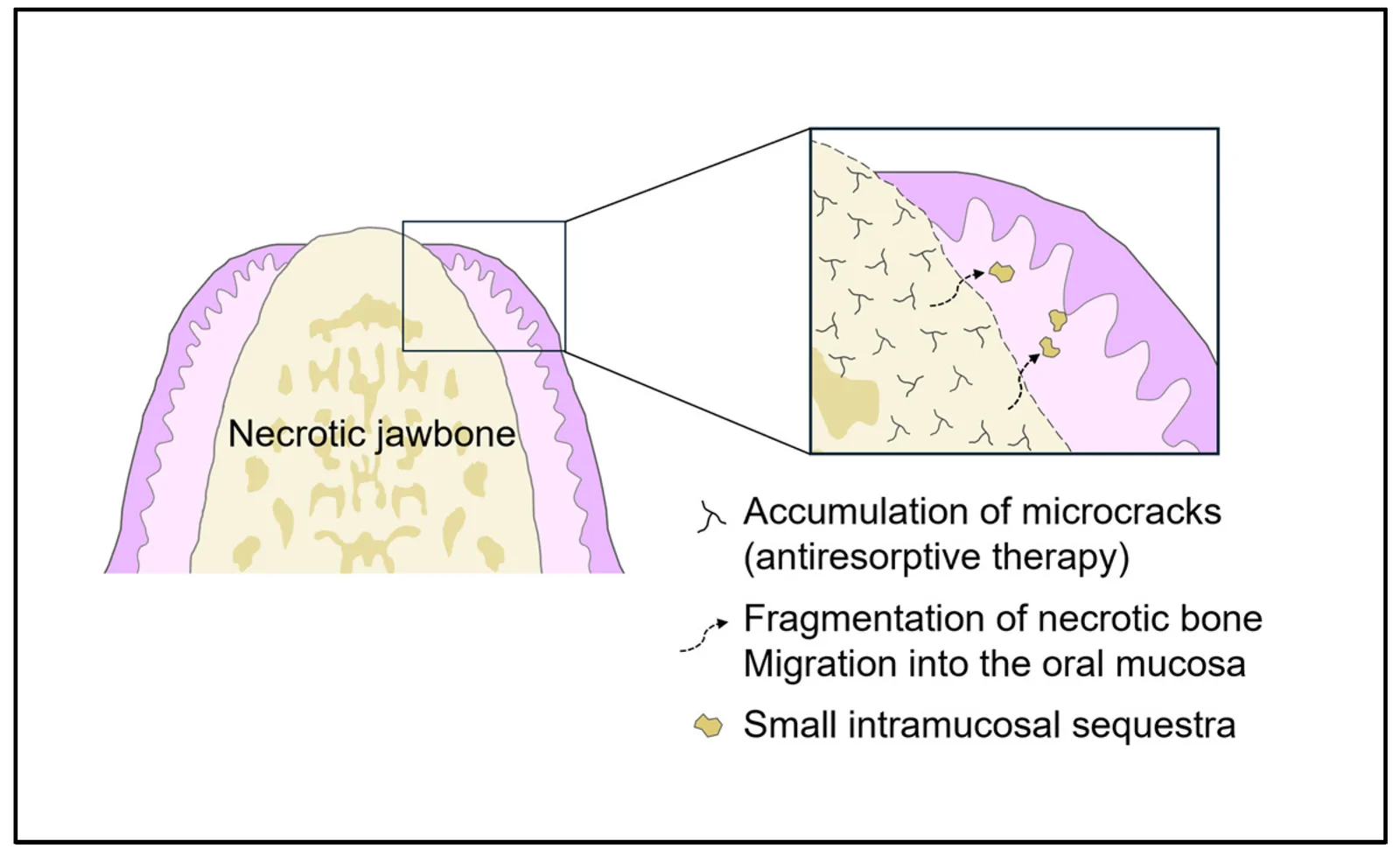
Schematic illustration of one hypothetical mechanism for the formation of small intramucosal sequestra in medication-related osteonecrosis of the jaw (MRONJ). In this proposed sequence, suppression of bone remodeling by antiresorptive therapy promotes the accumulation of microcracks within necrotic jawbone, leading to focal bone fragmentation and the generation of small devitalized bone particles. These particles subsequently migrate into the overlying oral mucosa. This sequence is speculative, and alternative mechanisms may include fragmentation secondary to advanced osteomyelitis or displacement of bone fragments along fistulous tracts.

The mechanisms by which small intramucosal sequestra develop remain uncertain. One possible explanation is that they originate from the surface of adjacent necrotic jawbone, although the processes responsible for their fragmentation and migration across the periosteum into the oral mucosa have yet to be elucidated. Antiresorptive agents suppress bone remodeling and impair the repair of microdamage caused by mechanical loading. Accordingly, previous studies have demonstrated increased microcrack formation in patients with bisphosphonate-related osteonecrosis of the jaw [12,13,14]. It is therefore conceivable that accumulation of microcracks and focal fragmentation of necrotic bone generate small devitalized bone particles that subsequently migrate into the overlying oral mucosa. However, this proposed pathway is speculative and cannot be established from the present histopathological observations. Alternative mechanisms, including fragmentation associated with severe inflammation or mechanical forces, may also contribute to the presence of bone fragments within the oral mucosa. One possible hypothetical mechanism is illustrated in Figure 5.

PEH was originally described in dermatopathology as a reactive epithelial proliferation that develops in response to persistent inflammation, chronic infection, or retained foreign material [15,16]. It has also been described in a variety of inflammatory disorders of the oral mucosa, including MRONJ [4,8,9,17,18,19]. In the present study, PEH was observed in all patients, which may reflect the advanced inflammatory changes associated with stage 3 MRONJ lesions. Direct contact between small intramucosal sequestra and the advancing epithelial fronts of PEH was observed in two of the six patients. In these cases, some sequestra were located adjacent to the advancing epithelial fronts, whereas others appeared to be located within the hyperplastic epithelium in the examined sections. However, because direct contact was observed in only two cases despite the presence of PEH in all patients, the association between small intramucosal sequestra and PEH should be interpreted cautiously.

A similar relationship was observed in our recent mouse study involving zoledronate administration followed by tooth extraction, in which small intramucosal sequestra developed in the healed oral mucosa covering extraction sockets, and some of these sequestra were observed in direct contact with, or within, PEH [20]. Based on these observations, we hypothesize that small intramucosal sequestra may be associated with a reactive epithelial response resembling that induced by retained foreign material. This hypothesis is of interest in light of the concept of transepithelial elimination, a well-recognized biological response in dermatopathology whereby foreign material is expelled through reactive epithelial proliferation [21,22]. Similar phenomena have also been described in oral tissues, including the transepithelial elimination of retained Alvogyl fibers from post-extraction tissues and of suture material through the labial mucosa [23,24]. Although the present findings are compatible with this possibility, the study was based on static cross-sectional histological sections and therefore cannot demonstrate epithelial migration or establish the temporal sequence of events. Alternative explanations, including passive incorporation of sequestra into proliferating epithelium or upward displacement of connective tissue during chronic inflammation, cannot be excluded. Whether a similar mechanism operates in the oral mucosa of patients with MRONJ warrants further investigation.

Several limitations should be acknowledged. First, the identification of the small sequestra were based on their appearance in individual two-dimensional histological sections and serial sectioning was not performed specifically to confirm three-dimensional isolation of all bone fragments. Therefore, the possibility that some fragments represented tissue-processing artifacts or remained continuous with the underlying jawbone cannot be completely excluded. Fragmentation secondary to advanced osteomyelitis and displacement of bone fragments along fistulous tracts also remain possible. Accordingly, the present findings do not establish their pre-existence in the mucosa before specimen handling. Further studies using systematic serial sectioning and three-dimensional reconstruction will be required to confirm their true spatial relationships. Second, this was a retrospective single-center case series including only six patients, all of whom had stage 3 mandibular MRONJ requiring extensive surgical resection. Therefore, the present findings should not be generalized to patients with earlier-stage disease or maxillary MRONJ. Because oral mucosal specimens suitable for histopathological evaluation can generally be obtained only from patients undergoing extensive surgical resection, and such cases are relatively uncommon, validation in larger cohorts will be necessary. Third, because no control group of other jawbone diseases was included, the specificity of small intramucosal sequestra for MRONJ cannot be determined from the present study. Similar findings could potentially occur in other conditions associated with jawbone necrosis or chronic inflammation, including osteoradionecrosis, infectious osteomyelitis, or aseptic osteonecrosis. Comparative studies including these diseases will therefore be necessary to establish whether small intramucosal sequestra represent a histopathological feature unique to MRONJ. Fourth, inflammatory cell phenotypes, cytokine expression, and other molecular mediators were not investigated. Further studies incorporating immunohistochemical markers of epithelial proliferation and inflammation, such as Ki-67, CD68, and TNF-α, together with molecular analyses, will be necessary to clarify the mechanisms underlying the association between small sequestra and epithelial responses.

In conclusion, small intramucosal sequestra were a recurrent histopathological finding in surgically resected oral mucosa from this selected series of patients with stage 3 mandibular MRONJ. These preliminary observations draw attention to histopathological changes in the oral mucosa overlying necrotic jawbone that have received limited attention. Further studies involving broader patient populations and appropriate comparison groups are required to validate this finding and determine its generalizability, disease specificity, and potential relevance to MRONJ pathogenesis.

## Figures and Tables

**Table 1:** Patient data.

Patient	Age	Sex	Underlying Disease	Antiresorptive Medication	Duration of Therapy	Lesion Site (Stage)	Surgical Procedure
1	61	M	CKD, OP	Zoledronate	6 years	Mandible (3)	Segmental
2	81	M	PC	Denosumab	6 years	Mandible (3)	Marginal
3	84	F	OP	Minodronate	1 year	Mandible (3)	Segmental
4	87	F	OP	Alendronate	5 years	Mandible (3)	Segmental
5	77	F	OP	Zoledronate	3 years	Mandible (3)	Segmental
6	61	F	BC	Denosumab	2 years	Mandible (3)	Marginal

CKD, chronic kidney disease; OP, osteoporosis; PC, prostate cancer; BC, breast cancer.

**Table 2:** Histopathological findings in oral mucosal specimens.

Patient	Inflammatory Cell Infiltration	PEH	Small Sequestra		
			Presence	Size (μm)	Contact
1	+	+	+	45–805	+
2	+	+	+	125–205	−
3	+	+	+	100–145	−
4	+	+	+	160–840	−
5	+	+	+	65–165	+
6	+	+	+	70–125	−

PEH, Pseudoepitheliomatous hyperplasia; contact, small sequestra in contact with PEH.

## Data Availability

The data presented in this study are available from the corresponding author upon reasonable request. The data are not publicly available because they contain information that could compromise the privacy of the research participants.

## Abbreviations

The following abbreviations are used in this manuscript:

MRONJ	Medication-related osteonecrosis of the jaw
PEH	Pseudoepitheliomatous hyperplasia
